# VEXAS syndrome: more than just vacuoles

**DOI:** 10.1016/j.htct.2025.103984

**Published:** 2025-09-16

**Authors:** Marco P. Barros Pinto

**Affiliations:** Hospital Santa Maria – Centro Hospitalar Universitário Lisboa Norte, EPE, Lisbon, Portugal; Faculdade de Medicina, Universidade de Lisboa, Lisbon, Portugal

A 78-year-old man with an autoimmune disorder (vasculitis) and a recent diagnosis of VEXAS syndrome, confirmed by next generation sequencing (presence of the p.Met41Thr variant of the *UBA1* gene with a variant allele frequency of 56.4 %) was admitted to hospital.

Analytically he presented: erythrocytes 3.75 × 10^12^/L (reference values [RV]: 4.5–5.9 × 10^12^/L), hemoglobin 132 g/L (RV: 130–175 g/L), mean corpuscular volume 103.6 fL (RV: 80–97 fL), leucocytes 3.9 × 10^9^/L (69 % neutrophils, 20 % lymphocytes), and platelets 197 × 10^9^/L (RV: 150– 450 × 10^9^/L).

The bone marrow aspirate smears were normocellular, with a myeloid to erythroid (M:E) ratio of 2:1. The smears also revealed 20 % myeloid precursors (all stages) and 30 % proerythroblasts with cytoplasmic vacuoles. Other notable findings included pseudo-Pelger-Hüet anomalies and megaloblastic precursors. Most of the megakaryocytes exhibited a high nuclear-to-cytoplasmic ratio; specifically, 50 % of total megakaryocytes (TM) were monolobated with eccentrically placed nuclei, and other megakaryocytes showed a wreath-like rearrangement of nuclear lobes. Additionally, multinuclear megakaryocytes (3 % TM) and megakaryocyte emperipolesis (3 % TM) were observed (Figure 1). There was no increase in blasts. Storage iron was decreased with no ring sideroblasts.

**Figure 1:**
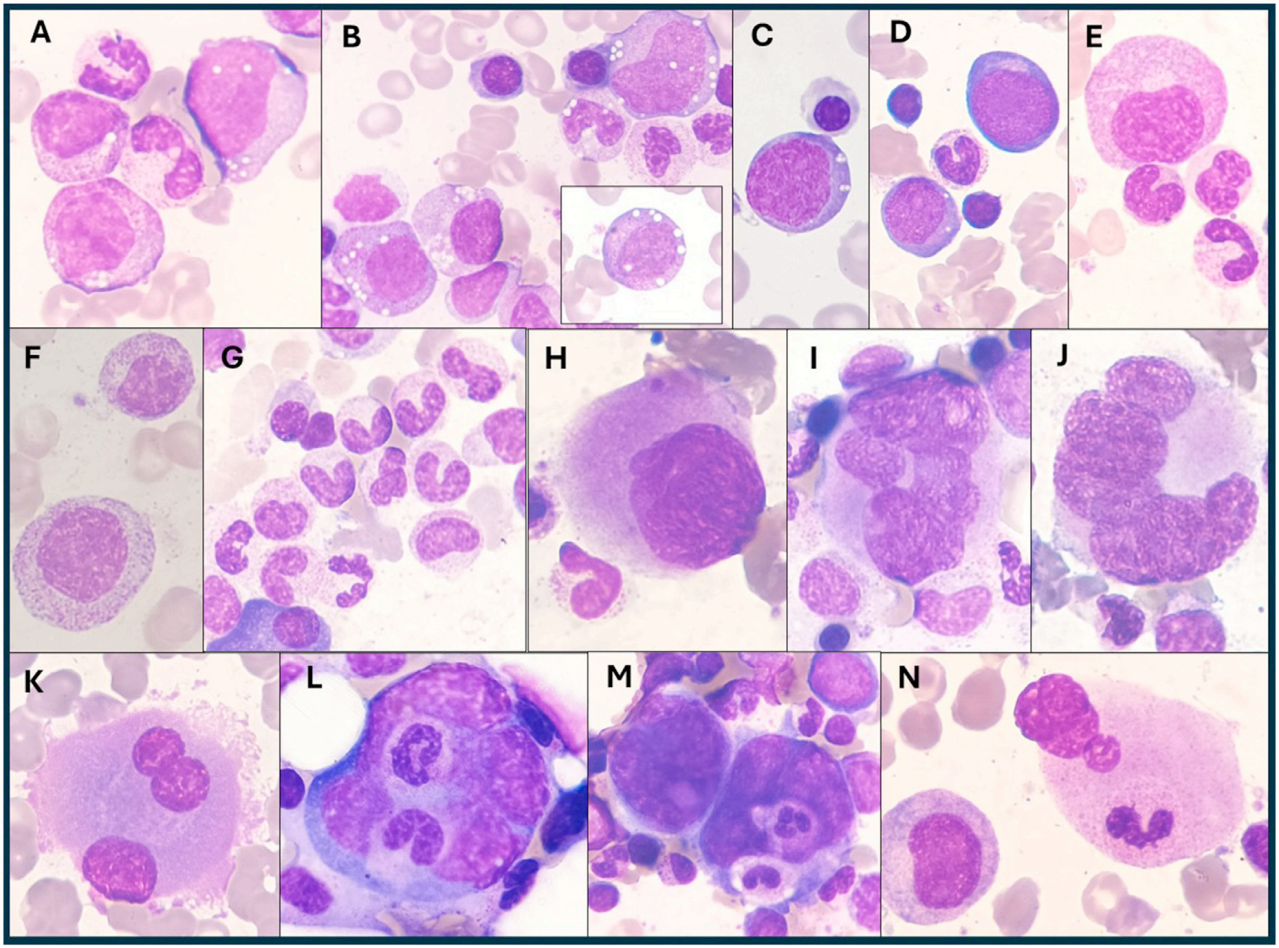
Bone marrow aspirate (May-Grünwald-Giemsa stain, × 100 objective): Myeloid precursors with vacuoles (A, B); proerythroblasts with vacuoles (C, D); megaloblastic precursors (D, E, F); pseudo-Pelger-Hüet (G); monolobated megakaryocytes (H); megakaryocytes with a wreath-like rearrangement of nuclear lobes (I, J); multinuclear megakaryocytes (K); megakaryocytes emperipolesis (L, M, N).

VEXAS syndrome (vacuoles, E1 enzyme, X-linked, autoinflammatory, somatic) was first reported by Beck et al. in 2020. Vacuoles are observed in erythroid and myeloid precursor cells [1]. The E1 enzyme is related to the ubiquitin activating enzyme encoded by the *UBA1* gene, an X-linked gene [1]. Mutation in this gene is responsible for an autoinflammatory disease (characterized by recurrent fevers, cytopenias, chondritis, vasculitis, pulmonary inflammation, and neutrophilic dermatoses) as the result of somatic mutations in the blood [1].

The most frequent mutations are p.Met41Thr (49 %), p.Met41Val (26 %) and p.Met41Leu (19 %) [2].

In VEXAS syndrome the bone marrow is usually hypercellular with an increased M:E ratio (>4:1 in >70 % of cases) [2]. The presence of ≥10 % of myeloid precursors with >1 vacuole can be both sensitive and specific for VEXAS syndrome, [3] however cytoplasmic vacuolization of myeloid and erythroid precursors can be found in other clinical settings: alcohol abuse, copper deficiency, treatments (chemotherapy and antibiotics), zinc toxicity, myelodysplastic syndrome, lymphoproliferative disorders, multiple myeloma, myeloproliferative neoplasms and acute myeloid leukemia, or as an artifact of sample preparation [2,3]. Furthermore, some atypical *UBA1* variants can present absence of precursor vacuolization in the bone marrow [2].

Storage iron is usually increased with no significant number of ring sideroblasts (<10 % of cases) [2].

The full blood count can show: macrocytic anemia (90–100 %), lymphopenia (60–80 %), monocytopenia (50 %), neutropenia (<30 %) and thrombocytopenia (45–69 %) [2].

The *UBA1* gene mutation is also a predisposing factor for myelodysplastic syndromes, plasma cell proliferation disorders (monoclonal gammopathy of undetermined significance, multiple myeloma) or both [2].

## Conflicts of interest

The author declares no conflicts of interest.
